# Supplementary material for: IL-1β turnover by the UBE2L3 ubiquitin conjugating enzyme and HECT E3 ligases limits inflammation
Source: Nat Commun. 2023 Jul 20;14:4385. doi: 10.1038/s41467-023-40054-x (PMC10359330; doi:10.1038/s41467-023-40054-x)
Supplement: Supplementary file 1 — Supplementary Information [file 41467_2023_40054_MOESM1_ESM.pdf]

IL-1β turnover by the UBE2L3 ubiquitin conjugating enzyme and HECT E3 ligases limits inflammation

Vishwas Mishra[1,*], Anna Crespo-Puig[1,*], Callum McCarthy[1], Tereza Masonou[1], Izabela Glegola-Madejska[1], Alice Dejoux[1], Gabriella Dow[1], Matthew J. G. Eldridge[1], Luciano H. Marinelli[1], Meihan Meng[1], Shijie Wang[1], Daniel J. Bennison[1], Rebecca Morrison[2], Avinash R. Shenoy[1,$]

[1] Medical Research Council Centre for Molecular Bacteriology & Infection, Imperial College London, London, UK

[2] Host-Pathogen Interactions in Tuberculosis Laboratory, The Francis Crick Institute, 1 Midland Road, London NW1 1AT, UK

* These authors contributed equally.

[$] Correspondence & Lead Contact:

Address: Room 4.40A, Flowers Bldg, Armstrong Road, Imperial College London, London SW7 2AZ, UK

Email: a.shenoy@imperial.ac.uk

Supplementary Figures S1-S5
Supplementary Tables S1-S2

Figure S1: *Mishra, Crespo-Puig et al*

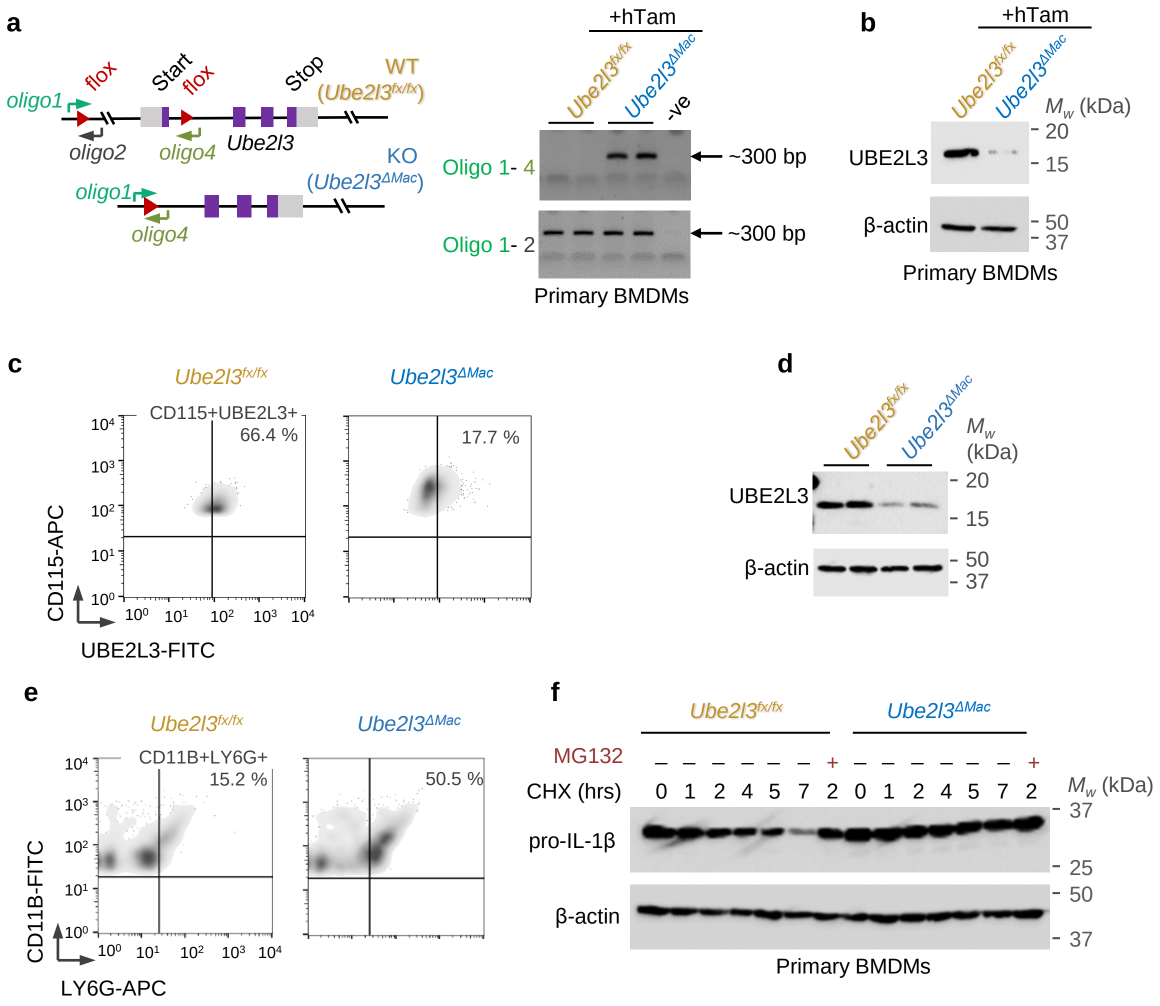

Figure S1. Generation of *Ube2l3*$^{\Delta Mac}$ mice. (a) Schematic depiction (left) and PCR-based screening (right) for genotyping of primary BMDMs from WT (*Ube2l3*$^{fx/fx}$) and KO (*Ube2l3*$^{fx/fx icre/Esr1}$ mice labelled *Ube2l3*$^{\Delta Mac}$ for convenience throughout) mice. Oligonucleotide (oligo) primer binding sites are indicated. Oligo 2 binding site is lost after Cre-mediated deletion of exon 1. Cells were treated with 4-hydroxytamoxifen (hTam, 2 µM) for 48 h before genomic DNA was prepared for PCR. Oligos 1 and 4 only generate a product if homologous recombination has occurred in cells expressing Cre (i.e., cells from KO mice), and no amplification is observed without Cre recombinase (i.e., WT cells). Oligos 1 and 2 generate a product at the "WT" locus in both genotypes, which indicates remnant WT alleles after hTam treatment in cells from KO mice. **(b)** Representative immunoblots of UBE2L3 and β-actin in cell lysates from primary BMDMs of the indicated genotypes given 4-hydroxytamoxifen (hTam, 2 µM, 48 h). **(c)** Representative flow cytometry density plot showing percentage of Csf1r/CD115 and UBE2L3+ve peritoneal macrophages isolated from mice of the indicated genotypes given tamoxifen (80 mg.mL^{-1}) orally on 3 consecutive days. WT = *Ube2l3*$^{fx/fx}$; KO = *Ube2l3*$^{\Delta Mac}$. **(d)** Representative immunoblots from peritoneal macrophages isolated from two mice each of the indicated genotypes given tamoxifen (80 mg.mL^{-1}) orally on three consecutive days. **(e)** Representative flow cytometry density plot showing percentage of CD11B and LY6G+ve cells in the peritoneum of mice treated with MSU. WT = *Ube2l3*$^{fx/fx}$; KO = *Ube2l3*$^{\Delta Mac}$. **(f)** Representative immunoblots of pro-IL-1β and β-actin in cell lysates from cycloheximide (CHX)-chase experiments carried out with primary BMDMs of the indicated genotypes given 4-hydroxytamoxifen (2 µM, 48 h). Cells were treated with LPS (250 ng.mL^{-1}) for 14 h followed by CHX (10 µg.mL^{-1}) for indicated times. MG132 (10 µM) was added for the last 3 h in the indicated samples. Each lane in **a-b** represents a mouse; data from independent experiments/mice as follows: **a**, n = 3; **b**, n = 4; **c**, n = 5 mice; **d**, n = 4 mice each; **e**, n = 8 mice; **f**, n = 2 experiments.

Figure S2: *Mishra, Crespo-Puig et al*

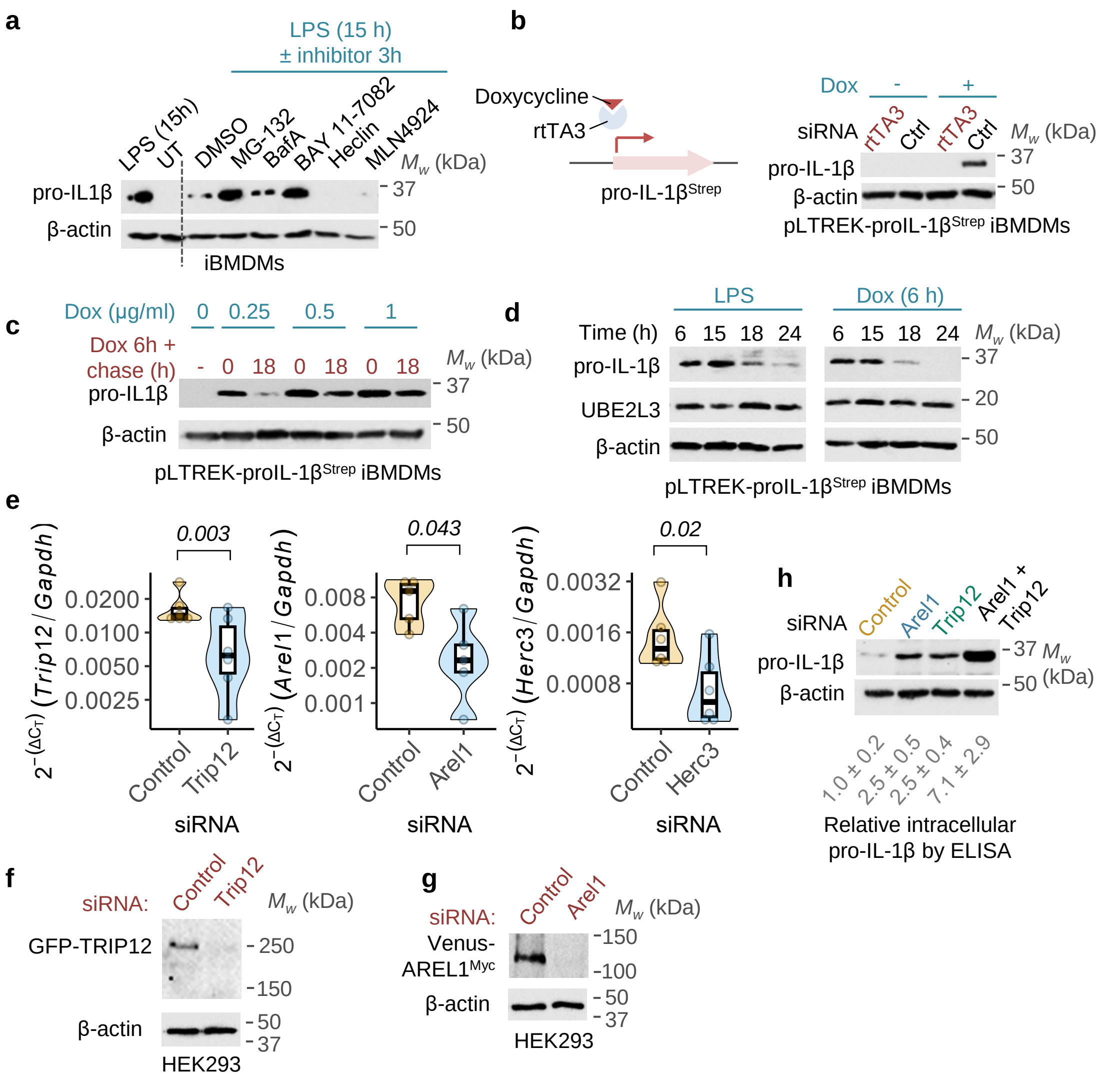

Figure S2: Validating the siRNA screening approach to identify E3 ligases involved in pro-IL-1β clearance. (a) Representative immunoblots showing the effect of the indicated inhibitors on the abundance of LPS-induced pro-IL-1β in iBMDMs. Cells were treated with LPS (250 ng.mL^{-1}) for 15 h or left untreated (UT) as controls (first two lanes, respectively). Other samples are from cells treated with LPS (250 ng.mL^{-1}) for a total of 18 h where the last 3 h included the indicated inhibitors or the solvent DMSO. **(b)** Schematic depiction (left) of rtTA3-transcription factor-dependent pro-IL-1β^{Strep} expression in pLTREK-proIL-1β^{Strep} iBMDMs cells. Representative immunoblots (right) from lysates of pLTREK-proIL-1β^{Strep} iBMDMs transfected with non-targeting control (Ctrl) or rtTA3 siRNA for 72 h, and then treated with doxycycline (Dox) for 6 h. **(c)** Representative immunoblots from pLTREK-proIL-1β^{Strep} iBMDMs treated with the indicated concentrations of Dox for 6 h, washed and incubated for either 0 or 18 h as labelled. **(d)** Representative immunoblots from pLTREK-proIL-1β^{Strep} iBMDMs treated with LPS (250 ng.mL^{-1}) or Dox (500 ng.mL-1) as indicated, followed by chase for the indicated times. **(e)** qRT-PCR showing the efficacy of silencing of *Arel1*, *Trip12* and *Herc3* transcripts. iBMDMs were transfected for 72 h with non-targeting control or siRNA against AREL1, TRIP12 or HERC3 as labelled. *Gapdh* was used as normalising control. **(f-g)** Representative images from experiments assessing the efficacy of siRNA on TRIP12 or AREL1 protein abundance. HEK293 cells were transfected with non-targeting Control or the indicated siRNA for 24 h, and then with plasmids encoding the indicated tagged proteins for an additional 24 h. **(h)** Representative immunoblots from experiments assessing the combined effect of TRIP12 and AREL1 silencing. iBMDMs were transfected with the indicated siRNA for 72 h followed by treatment with LPS (250 ng.mL^{-1}) for 24 h (n = 3 experiments). Numbers below (mean ± SEM) are intracellular pro-IL-1β measured by ELISA normalised to Control siRNA samples (n = 3-9).

Images in **a-d, f-h** represent experiments performed at least n = 3 times. In **e** each dot represents a biologically independent experiment (n = 5). Data distribution is depicted with violin, box (25th to 75th percentile, line at median), and whiskers (± 1.5 x IQR). Two-tailed *P* values for indicated comparisons from linear mixed effects models.

Figure S3: *Mishra, Crespo-Puig et al*

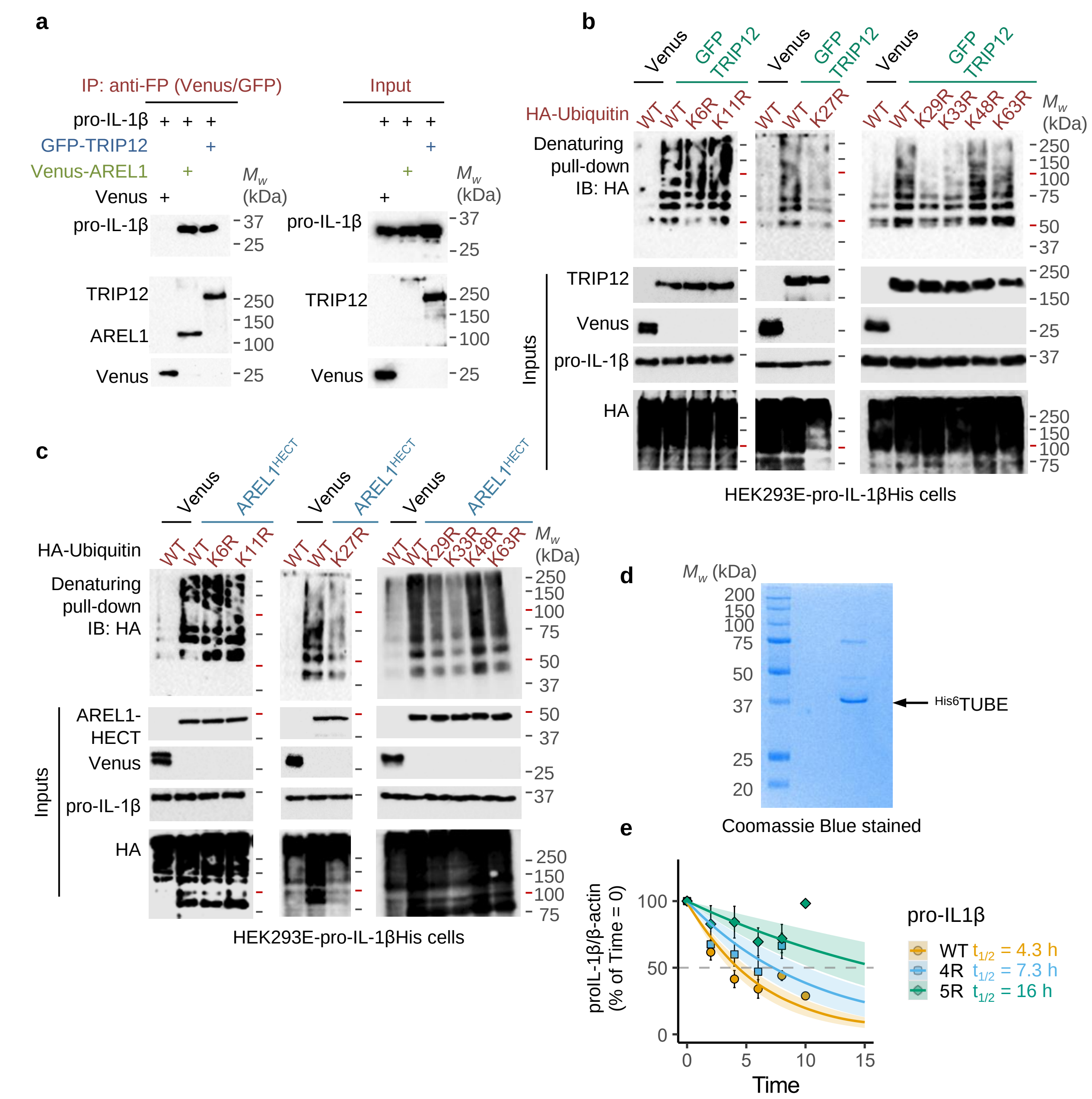

Figure S3. AREL1 and TRIP12 ubiquitylate pro-IL-1β in the 'pro' domain. (a) Representative images of immunoprecipitation (IP) and immunoblot (IB) experiments to assess the interaction between pro-IL-1β and TRIP12 or AREL1. HEK293E cells were transfected with plasmids encoding Venus-AREL1^{Myc} or GFP-TRIP12 or Venus as negative control followed by IP with anti-FP (fluorescent protein) antibody and IB with pro-IL-1β or FP antibodies as labelled. Anti-FP (fluorescent protein) antibody detects both GFP and Venus tags. Expression of proteins is shown on right (Input). **(b-c)** Representative immunoblots from Ni-NTA pull-downs of pro-IL-1βHis under denaturing conditions (8 M urea-containing buffers) to assess the type of ubiquitin chains covalently added on pro-IL-1β by TRIP12 (**b**) or AREL1 (**c**). HEK293E cells stably expressing pro-IL-1βHis were transfected with HA-tagged wildtype or the indicated K→R mutants of ubiquitin along with GFP-TRIP12 (**b**) or AREL-HECT^{Myc} (**C**) or Venus as negative control. Expression of proteins is shown below (Inputs). **(e)** SDS-PAGE of purified recombinant ^{His6}TUBE (~2 µg) followed by Coomassie staining. **(e)** Relative rate of turnover of transiently expressed indicated variants of pro-IL-1β in HEK293 cells. pro-IL-1β and β-actin were quantified from immunoblots following CHX-chase experiments (similar to those in Figure 3b), and the percentage of pro-IL-1β normalised to β-actin relative to Time = 0 h is plotted. The half-life ($t_{1/2}$, indicated by dotted line at 50 %) of pro-IL-1β in the two genotypes was calculated by fitting a first-order exponential decay curve. Shaded regions indicate 95 % confidence interval of the fit.

Images in **a-c** from experiments performed at least n = 3 times; **d**, n = 2; **e**, n = 8 (mean ± SEM error bars are shown).

Figure S4: *Mishra, Crespo-Puig et al*

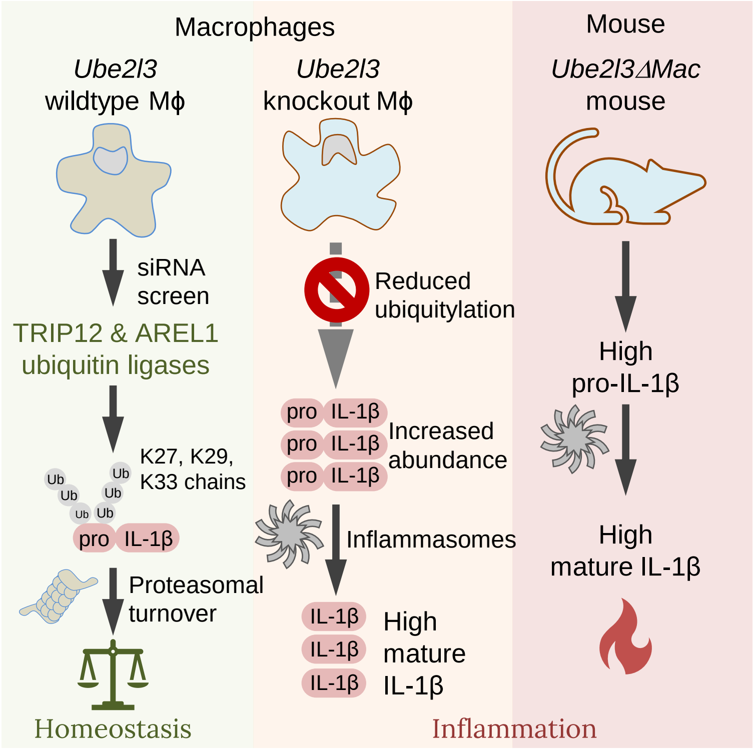

Figure S4. Model of pro-IL-1β turnover by UBE2L3 ubiquitin conjugating enzyme, and TRIP12 and AREL1 HECT ubiquitin ligases. In wildtype macrophages, pro-IL-1β protein undergoes natural turnover by its ubiquitylation by TRIP12 and AREL1 ubiquitin E3 ligases of the HECT (homologous to E6AP C-terminus) subfamily. These ligases add unconventional K27-, K29- and K33- poly-ubiquitin chains on lysine residues mainly in the 'pro' domain of pro-IL-1β. Deletion of *Ube2l3* or silencing *Trip12* or *Arel1* in macrophages increases pro-IL-1β abundance. Increased pro-IL-1β becomes available for proteolytic maturation by caspase-1 inflammasomes leading to increased bioactive IL-1β production. *Ube2l3*$^{\Delta Mac}$ mice secrete high IL-1β and display signs of inflammation *in vivo* upon inflammasome activation.

Figure S5: *Mishra, Crespo-Puig et al*

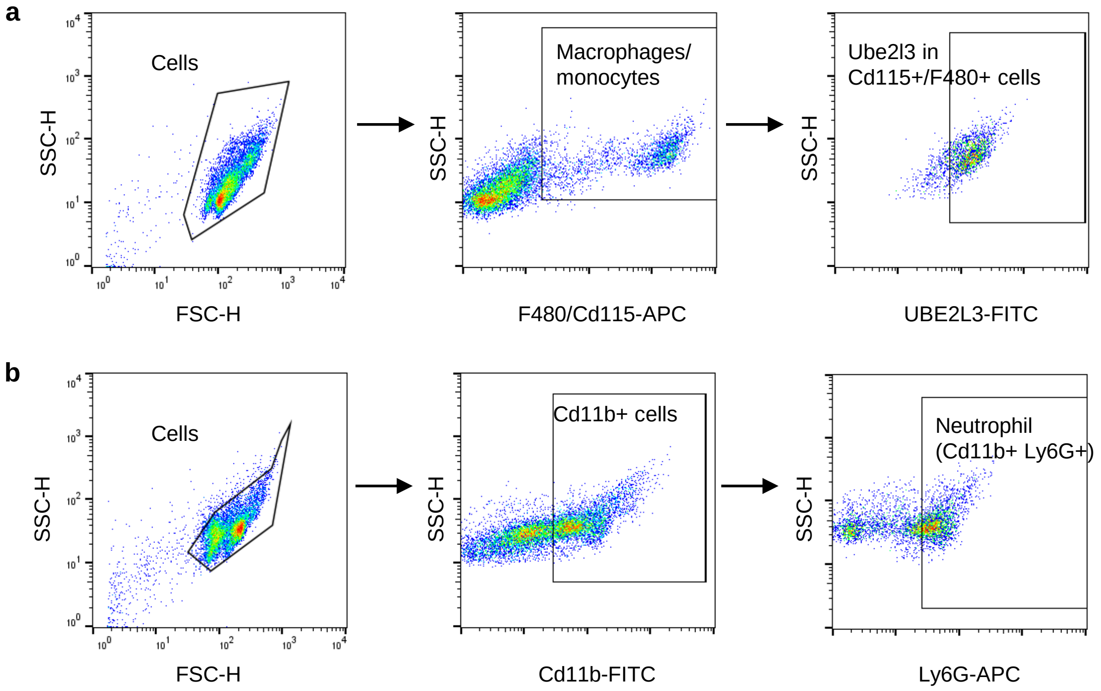

Figure S5. Gating strategy for flow cytometry. (a) Gating strategy for flow cytometric analysis of intracellular UBE2L3 levels in CD115 or F4/80+ cells (i.e., results in ***Fig 1B-E, 2B, S1C).*** **(b)** Gating strategy for flow cytometric analysis of number of Cd11b+ Ly6G+ neutrophils in the peritoneal cavity of MSU treated mice (i.e., results in ***Fig 2J, S1E***).

Supplementary Table S1

Antibody	Company	Catalogue number	Antibody dilution	Antibody validation in the laboratory or by commercial vendor (text and links from websites).
Anti-CD115-APC	Biolegend	Cat# 135509	1:100	Each lot of this antibody is quality control tested by immunofluorescent staining with flow cytometric analysis. For flow cytometric staining, the suggested use of this reagent is ≤ 0.25 µg per 10^6 cells in 100 µl volume. It is recommended that the reagent be titrated for optimal performance for each application. https://www.biolegend.com/en-us/products/apc-anti-mouse-cd115-csf-1r-antibody-6336?GroupID=BLG8949
Anti- F4/80 Antigen APC	Tonbo Biosciences	Cat# 20-4801-U025	1:100	This antibody preparation has been quality-tested for flow cytometry using mouse spleen cells, or an appropriate cell type (where indicated). https://cytekbio.com/products/apc-anti-mouse-f4-80-antigen-bm8-1?variant=40581236424740
Anti- GSDMD	Abcam	Cat# ab209845	1:1000	Knockout validated https://www.abcam.com/products/primary-antibodies/gsdmd-antibody-epr19828-ab209845.html
Anti-ASC (AL177)	Adipogen	Cat# AG-25B-0006-C100	1:1000	We have validated this antibody against *Asc*$^{-/-}$ iBMDMs.
Anti-Caspase-1 (Casper1)	Adiopgen	Cat# AG-20B-0042-C100	1:1000	We have validated against *Casp1/11*$^{-/-}$ iBMDMs. Recognizes endogenous full-length and activated (p20 fragment) mouse caspase-1. Described to cross-react with full-length and activated (p20 fragment) of rat caspase-1 . https://adipogen.com/ag-20b-

				0042-anti-caspase-1-p20-mouse-mab-casper-1.html
Anti-Cd11b-FITC	Biolegend	Cat# 101206	1:100	Each lot of this antibody is quality control tested by immunofluorescent staining with flow cytometric analysis. https://www.biolegend.com/en-us/products/fitc-anti-mouse-human-cd11b-antibody-347?GroupID=BLG10660
Anti-CD16/32	Biolegend	Cat# 101302	1:100	Each lot of this antibody is quality control tested by immunofluorescent staining with flow cytometric analysis. https://www.biolegend.com/en-us/products/purified-anti-mouse-cd16-32-antibody-190
Anti-c-Myc (9E10)	SCBT	Cat# sc-40	1:3000	This Myc antibody is recommended for detection of c-Myc p67 and c-Myc tagged fusion proteins of mouse, rat, human, monkey, feline and canine origin by WB, IP, IF, IHC(P), FCM and ELISA; non cross-reactive with N-Myc or L-Myc proteins. Widely used in combination with eukaryotic expression vectors encoding proteins with c-Myc (amino acids 408-439) epitope tag. https://www.scbt.com/p/c-myc-antibody-9e10
Anti-Flag M2 antibody	Sigma-Aldrich	Cat# F1804	1:2000	Optimized for single banded detection of FLAG fusion proteins in mammalian, plant, and bacterial expression systems. https://www.sigmaaldrich.com/GB/en/product/sigma/f1804
Anti-FP (i.e., anti-GFP/mVenus/AcGFP) mouse monoclonal	Roche	Cat# 11814460001	1:2000	Anti-GFP is a mixture of two clones (7.1 and 13.1). Monoclonal antibody for detection of both wild-type and mutant forms of GFP or GFP fusions using: Immunoprecipitation, western blots.

				https://www.sigmaaldrich.com/GB/en/product/roche/11814460001
Anti-GAPDH	Sant Cruz	Cat# sc-365062	1:2000	Molecular Weight of GAPDH: 37 kDa.<br>Positive Controls: Hep G2 cell lysate: sc-2227, HeLa whole cell lysate:<br>sc-2200 or A549 cell lysate: sc-2413.<br>https://www.scbt.com/p/gapdh-antibody-g-9?requestFrom=search
Anti-IL-1β/IL-1F2 Goat polyclonal Antibody	R&D systems	Cat# AF401	1:2000	Detects mouse IL-1 beta /IL-1F2 in direct ELISAs and Western blots.<br>https://www.rndsystems.com/products/mouse-il-1beta-il-1f2-antibody_af-401-na
Anti-Ly-6G-APC	Biolegend	Cat# 127614	1:100	Each lot of this antibody is quality control tested by immunofluorescent staining with flow cytometric analysis.<br>https://www.biolegend.com/en-us/products/apc-anti-mouse-ly-6g-antibody-6115
Anti-NLRP3 (Cryo2)	Adipogen	Cat# AG-20B-0014-C100	1:1000	We have validated this antibody against *Nlrp3*$^{-/-}$ cells.
Anti-UBE2L3	GeneTex	Cat# GTX104717	1:3000	We have tested this antibody against knockout or siRNA transfected cells.
Anti-UBE2L3 (B-11) FITC	Santa Cruz	Cat# sc-390032	1:100	We have tested this antibody against knockout or siRNA transfected cells.
Anti-β-Actin−Peroxidase	Sigma-Aldrich	Cat# A3854	1:10000	western blot: 1:25,000-1:50,000 using cell extracts of human foreskin fibroblasts or chicken fibroblasts.<br>https://www.sigmaaldrich.com/GB/en/product/sigma/a3854
Donkey anti-Goat IgG(H+L) Secondary Antibody,	Thermo Fisher Scientific	Cat# A16005	1:20000	Based on Immunoelectrophoresis, no reactivity is observed to: non-immunoglobulin goat serum immunoglobulins, IgG from human, mouse, rabbit or rat.

HRP conjugate				https://www.thermofisher.com/antibody/product/Donkey-anti-Goat-IgG-H-L-Cross-Adsorbed-Secondary-Antibody-Polyclonal/A16005
Donkey anti-Rabbit IgG (H+L) Secondary Antibody, HRP conjugate	Thermo Fisher Scientific	Cat# A16035	1:20000	Based on Immunoelectrophoresis, no reactivity is observed to: non-immunoglobulin rabbit serum proteins, IgG from bovine, chicken, goat, guinea pig, hamster, horse, human, mouse, rat or sheep. https://www.thermofisher.com/antibody/product/Donkey-anti-Rabbit-IgG-H-L-Highly-Cross-Adsorbed-Secondary-Antibody-Polyclonal/A16035
Goat anti-Mouse IgG (H+L) Highly Cross-Adsorbed Secondary Antibody HRP	Thermo Fisher Scientific	Cat# A16078	1:20000	Based on Immunoelectrophoresis, no reactivity is observed to: non-immunoglobulin mouse serum proteins, bovine, goat, human, rabbit or rat IgG. https://www.thermofisher.com/antibody/product/Goat-anti-Mouse-IgG-H-L-Highly-Cross-Adsorbed-Secondary-Antibody-Polyclonal/A16078
Normal mouse IgG2α FITC	Santa Cruz	Cat# sc-2856	1:100	recommended for use as a negative control immunoglobulin in place of a target specific primary antibody of the same isotype (mouse IgG2a) by IF, IHC(P) and FCM applications. https://www.scbt.com/p/normal-mouse-igg2a-fitc?requestFrom=search
Rat IgG2α k - APC isotype	Biolegend	Cat# 400511	1:100	Each lot of this antibody is quality control tested by immunofluorescent staining with flow cytometric analysis as negative control. https://www.biolegend.com/en-gb/products/apc-rat-igg2a-kappa-isotype-ctrl-1838

Supplementary Table S2

REAGENT or RESOURCE	SOURCE	IDENTIFIER
**Chemicals and Recombinant Proteins**		
Acrylamide (40%)	Fisher Bioreagents	Cat # BP1408-1
Acrylamide (40%) 37.5:1	Sigma-Aldrich	Cat # A7168
Adenosine 5′-triphosphate (ATP) disodium salt	Sigma-Aldrich	Cat # A2383
Bafilomycin A1 from *Streptomyces griseus*	Sigma-Aldrich	Cat # B1793
BAY 11-7082	Cayman Chemicals	Cat # CAY10010266
BSA	Sigma-Aldrich	Cat # A3059
cOmplete protease inhibitor cocktail	Roche	Cat# 04693116001
Cycloheximide	Sigma-Aldrich	Cat # C7698
DMSO	Sigma-Aldrich	Cat # D2438-50ML
Doxycycline	Sigma-Aldrich	Cat # 9891
Dulbecco's minimal Eagle media High Glucose (4500mg/L)	Sigma-Aldrich	Cat# D5796
Dulbecco's minimal Eagle media Low Glucose (1000mg/L)	Sigma-Aldrich	Cat# D6046
Dulbecco's PBS	Sigma-Aldrich	Cat # D8537
Fetal Bovine Serum	Sigma-Aldrich	Cat# F9665
Gentamicin	Sigma-Aldrich	Cat # G1272
Heclin	Sigma-Aldrich	Cat # SML1396
IPTG	Sigma-Aldrich	Cat # I6758
Lipofectamine™ 2000 Transfection Reagent	Thermo Fisher Scientific	Cat # 11668030
LPS-EB (ultra-pure LPS from E. coli O111:B4)	Invivogen	Cat# tlrl-3pelps
Luria broth (LB)	Sigma-Aldrich	Cat # L3022
MG-132	Calbiochem	Cat # 474787
MLN4924	MedChemexpress	Cat # HY-10484
Monosodium uric acid crystals	Invivogen	Cat # tlrl-msu-25
Nigericin sodium salt	Sigma-Aldrich	Cat # N7143
Non-Fat Dried Milk	VWR	Cat # A0830.1000
NP-40	AppliChem	Cat # A1694
Opti-MEM	Gibco	Cat#31985062
PBS 10x	Fisher	Cat #10214733
Penicillin-Streptomycin	Sigma-Aldrich	Cat# P4333
Pierce™ Phosphatase Inhibitor Mini Tablets	Thermo Fisher Scientific	Cat# A32957
Pierce™ Protease Inhibitor Mini Tablets, EDTA-free	Thermo Fisher Scientific	Cat# A32955

PMSF	Sigma-Aldrich	Cat# P7626
Polyethylenimine (PEI)	Sigma-Aldrich	Cat # 408700
Precision Plus Protein Dual Color Standards	Bio-Rad	Cat # #1610374
Propidium Iodide	Sigma-Aldrich	Cat # P4170
PVDF membrane	Bio-Rad	Cat # 1620177
RNAse away	VWR	Cat # 732-2271
Sodium pyruvate	Sigma-Aldrich	Cat# S8636
SYBR Safe DNA gel stain	Life Technologies	Cat # S43102
Tamoxifen	Sigma-Aldrich	Cat # A5648
TEMED	Sigma-Aldrich	Cat # T9281
Thioglycolate	Sigma-Aldrich	Cat # 70157
TransIT-X2® Dynamic Delivery System	Mirus	Cat # MIR 6004
Triton	VWR Chemicals	Cat # 9002-93-1
Trypan Blue	Sigma-Aldrich	Cat # T8154
Trypsin-EDTA solution-100ml	Sigma-Aldrich	Cat# T4049
Tween 20	Sigma-Aldrich	Cat # P1379
**Critical Commercial Kits**		
Clarity Western ECL Blotting substrate	Bio-Rad Laboratories	Cat# 1705061
E.Z.N.A Total RNA kit I	Omega Bio-tek	Cat # R6834-02
ECL™ Prime Western Blotting Detection Reagent	GE-Healthcare	Cat# RPN2236
Gel extraction kit	NEB	Cat # T1020
High-capacity cDNA Reverse Transcription kit	Thermo Fisher Scientific	Cat # 4368814
LookOut® Mycoplasma PCR Detection Kit	Sigma-Aldrich	Cat # MP0035
KOD Hot-start polymerase	Merck	Cat # 71086
Mini prep Kit (200 tests)	VWR OMEGA	Cat # D6943-02
mouse IL-1β ELISA kit	Thermo Fisher Scientific	Cat # 88-7013-88
mouse IL-6 ELISA kit	Thermo Fisher Scientific	Cat # 88-7064-88
mouse pro-IL-1β ELISA kit	Thermo Fisher Scientific	Cat # 88-8014-22
mouse TNFα ELISA kit	Thermo Fisher Scientific	Cat # 88-7324
Phusion High-Fidelity DNA Polymerase	New England Biolabs	Cat # M0530
SoAdvanced Universal SYBR green supermix	Bio-Rad Laboratories	Cat # 1725270
SYBR™ Green PCR Master Mix	Thermo Fisher Scientific	Cat # 4309155

TaqMan Reverse Transcription Reagents	Thermo Fisher Scientific	Cat # N8080234
**Bacterial and virus strains**		
BL21-CodonPlus (DE3)-RIPL	52	
**Experimental Model: Cell Lines**		
HEK293E	52	
iBMDM	6	
**Recombinant DNA**		
pLTREK-2P-3Flag-mIL1β-2xStreptac	This study	Modified from pLTREK-TEV-T2A-GFP [53]
pLTREK-2P-mIL1β-2xStreptac	This study	
pLTREK-2P-mIL1β	This study	
pLTREK3-TEV-T2-GFP	53	
pMX-CMV-pro-IL-1βHis	This study	
pMX-CMV-mIL1b_2Strep	This study	
pMX-CMV-YFP_Arel1	This study	
pMX-CMV-AREL1-3Myc	This study	
pMX-CMV-pro-IL-1βstrep-5R	This study	
pMX-CMV-pro-IL-1βstrep-4R	This study	
pMX-CMV-mVenus-AREL1-2Myc-C790A	This study	
pMX-CMV-AREL1-2Myc-C790A	This study	
pCMV6-Mouse Arel1 E3 ligase-Myc	Origene	Cat # MR210828
pAcGFP-TRIP12	56	Jiri Lukas
pMx-CMV-YFP	6	
pMxCMV-YFP-UBE2L3	6	
pMX-CMV-mVenus-2Myc	This study	
pMX-CMV-2Myc	This study	
pRSET-6xTR-TUBE	20	Addgene Cat #110313
pPRO_Ex-HIST-TEV_6xTR-TUBE	This study	
pCMV-VSV-G	6	Walther Mothes
pCMV-MMLV	6	Walther Mothes
pCMV-HIV-Gag-Pol	6	Walther Mothes
HA-ubiquitin variant K6R	66	Prof. Hiroshi Ashida
HA-ubiquitin variant K11R	66	Prof. Hiroshi Ashida
HA-ubiquitin variant K27R	66	Prof. Hiroshi Ashida
HA-ubiquitin variant K29R	66	Prof. Hiroshi Ashida
HA-ubiquitin variant K33R	66	Prof. Hiroshi Ashida
HA-ubiquitin variant K48R	66	Prof. Hiroshi Ashida
HA-ubiquitin variant K63R	66	Prof. Hiroshi Ashida
**Oligonucleotides**		
RTPCR_Il1b_F	6	
CTACCTGTGTCTTTCCCGTG		
RTPCR_Il1b_R	6	
TGCAGTTGTCTAATGGGAACG		
RTPCR_Arel1_F	This study	

GATGGAGCTGGTCTTTGCAG		
RTPCR_Arel1_R	This study	
GCTCCACCTCCTCTTTCACT		
RTPCR_Trip12_F	This study	
TTAAGCGCACAGTCCAACAG		
RTPCR_Trip12_R<br>TGTGCCTGCTCCTTAATCCA	This study	
RTPCR_Herc3_F	This study	
GTTCACGAGTGGTACACAGC		
RTPCR_Herc3_R	This study	
TCCAATGGGAACTCGTGGAA		
RTPCR_Ube2l3_F	This study	
GGGCATCAAAAGACCAAAGCA		
RTPCR_Ube2l3_R	This study	
GCTAGAACCCCCTCACTTCC		
mProIL1b-pLPP-BamHI-rvs	This study	
AATTGAGGATGACTCCAggatccGGAAGACACGGATTCCATG		
mIL1b-NT-stop-NheI-pLTRE-rvs	This study	
cttgtacagctcgtccatGCTAGCTCAGGAAGACACGGATTCCATGG		
mIL1b-NT_KzEcoRV-pLTRE-fwd	This study	
ccgtcagatcgcctgatatcGCCACCATGGCAACTGTTCCTGAACTC		
pLTREK_pro-IL1-rvs	This study	
cggagccacctcctttttcgaattgaggatgactccaggatcGTCACACACCAGCAGGTTATCATCATCA		
pMX_mature_mIL1_AfeI-frw	This study	
accgtcagatccgctagcgctagccaccatggGTTCCCATTAGACAACTGCA		
pMX_mIL1b_2Strp_EcoRI_rv	This study	
GCCGGCCCTCGAGGCCTGCAGGAATtcacttctcaaactgcgggtg		
pMX_mIL1b_AfeI_fw<br>CCGTCAGATCCGCTAGCGCTAGCCACCatggcaactgttcctgaactca	This study	
pMX_pro-IL1-2Strep_BstBI_rvs	This study	
tcgaattgaggatgactccaggatccACACACCAGCAGGTTATCATCAT		
pMXYFPmIL1full_fw	This study	
GTCGACGGTACCGCGGGCCCGatggcaactgttcctgaact		
mIL1b_K133R_HindIII-fwd	This study	
GATGAACAACAAAgaAGCtTaGTGCTGTCGGAC		

mIL1b_K133R_HindIII-rvs	This study	
GTCCGACAGCACtAaGCTtcTTTGTTGTTCATC		
mIL1b_K30R-K32R_SphI-fwd	This study	
GAAGTTGACGGACCCCAAcgcATGcgGGGCTGCTTCCAAACCTTT		
mIL1b_K30R-K32R_SphI-rvs	This study	
AAAGGTTTGGAAGCAGCCCcgCATgcgTTGGGGTCCGTCAACTTC		
mIL1b_K58R_HindIII_F	This study	
CTCGCAGCAGCACATCAACcgaAGCTTCAGGCAGGCAGTAT		
mIL1b_K58R_HindIII_R	This study	
ATACTGCCTGCCTGAAGCTtcgGTTGATGTGCTGCTGCGAG		
mIL1b-K72R-AfeI-rvs	This study	
GACACAGGTAGCTGCCACAagcgCTCCACAGCCACAATGAGT		
mIL1b-K72R-AfeI-fwd	This study	
ACTCATTGTGGCTGTGGAGcgctTGTGGCAGCTACCTGTGTC		
mIL1b-6H-Pme-fwd	This study	
CACCATCACCATCACTAGTTTAAACGAATTCCTGCAGGCCTCGAG		
mIL1b-6H-Pme-rvs	This study	
GTTTAAACTAGTGATGGTGATGGTGATGACTCCAGGATCCGGAAGAC		
AREL1_I424T-SnaBI-fwd	This study	
GCAGCCACTTTCATACGTAGCTTGCACAAGAACATTGGAGG		
AREL1_I424T-SnaBI-rvs	This study	
TGTTCTTGTGCAAGCTACGTATGAAAGTGGCTGCTAGGATATT		
AREL1-KzAfeI-fwd	This study	
ACCGTCAGATCCGCTAGCGCTAGCCACCATGTTTTACGTTATTGGTGGAATCAT		
AREL1-2Myc-BamHI-rvs	This study	
GAGATCAGCTTCTGCTCGGATCCGAGCATGCCAAAGCCCTCG		
AREL1_436-AfeI-fwd	This study	
ACCGTCAGATCCGCTAGCGCTAGCCACCATGTCTGAGACCTTTCAAGACAAGG		
allFP_AfeI-fwd	This study	
AACCGTCAGATCCGCTAGCGCTGCCACCATGGTGAGCAAGGGC		
mVen-AREL1-ol-rvs	This study	

GTAAAACATGGTGGCTAGCCCCTTGTA CAGCTCGTCCATGCCGA		
**Software**		
BD FACStation (v6.1)	BD Biosciences	https://www.bdbiosciences.com
Cyflogic (v1.2.1)	CyFlo Ltd	http://www.cyflogic.com/index.php
FlowJo (v10.8.1)	BD Biosciences	https://www.bdbiosciences.com
ggbeeswarm (v0.7.1 and above)	CRAN	https://cran.r-project.org/package=ggbeeswarm
ggResidpanel (v0.3.0)	CRAN	https://CRAN.R-project.org/package=ggResidpanel
ggplot2 (v3.3.0 and above)	CRAN	https://CRAN.R-project.org/package=ggplot2
grafify (v2.0 and above)	CRAN	https://CRAN.R-project.org/package=grafify
GraphPad Prism (v8.0 and above)	GraphPad Software	https://www.graphpad.com/scientific-software/prism/
Image Lab (v6.0.1)	Bio-Rad	http://www.bio-rad.com/en-uk/product/image-lab-software?ID=KRE6P5E8Z
lme4 (v1.1-25 and above)	CRAN	https://CRAN.R-project.org/package=lme4
lmerTest (v3.1-2 and above)	CRAN	https://CRAN.R-project.org/package=lmerTest
MARS (v4.01)	BMG Labtech	https://www.bmglabtech.com/en/microplate-reader-software/
Microsoft Office 365 Enterprise	Microsoft	https://www.office.com
Omega (v5.5)	BMG Labtech	https://www.bmglabtech.com/en/microplate-reader-software/
performance (v0.4.3 and above)	CRAN	https://CRAN.R-project.org/package=performance
R (v4.0 and above)	The Comprehensive R	https://cran.ma.imperial.ac.uk/

	Archive Network (CRAN)	
RStudio Desktop (v1.3 and above)	RStudio	https://www.rstudio.com/products/rstudio/#rstudio-desktop
StepOne (v2.3)	Applied Biosystems	https://www.thermofisher.com/uk/en/home/life-science/pcr/real-time-pcr/real-time-pcr-instruments/step-one-real-time-pcr-systems.html
**siRNA ON-TARGETplus SMARTpool**		
siRNA ON-TARGETplus non-targeting	Dharmacon	D-001810-10
- UGGUUUACAUGUCGACUAA -		(D-001810-01)
- UGGUUUACAUGUUGUGUGA -		(D-001810-02)
- UGGUUUACAUGUUUUCUGA -		(D-001810-03)
- UGGUUUACAUGUUUUCCUA -		(D-001810-04)
siRNA Arel1	Dharmacon	
CAUCAAUAAUGGCGAAUUU		J-059761-09
GAAGAAUAUUGUUGAACGA		J-059761-10
CCAUGGAGGGAGAACGCUA		J-059761-11
CGAAGAAGGUGUACUGCUA		J-059761-12
siRNA Trip12	Dharmacon	
CGAAUCAACUGGUGCCGAA		J-053913-09
CGGCAGAGAGAUCCGGUUA		J-053913-10
UCGCAAAGGUUAAGAUGAA		J-053913-11
CGCCUAGAUUGGAUAGAAA		J-053913-12
siRNA HERC3	Dharmacon	
GCAGACUGAUGCCGAGUUA		J-055904-09
AAGAAUUCGUGGACGCUUA		J-055904-10
UGAUGAAGUUAAUCCGAGA		J-055904-11
CAGAACACAACAACGCAAA		J-055904-12
