# Supplementary material for: IL-1β turnover by the UBE2L3 ubiquitin conjugating enzyme and HECT E3 ligases limits inflammation
Source: Nat Commun. 2023 Jul 20;14:4385. doi: 10.1038/s41467-023-40054-x (PMC10359330; doi:10.1038/s41467-023-40054-x)
Supplement: Supplementary file 3 — Reporting Summary [file 41467_2023_40054_MOESM3_ESM.pdf]

nature portfolio

Corresponding author(s): Avinash R. Shenoy

Last updated by author(s): Jun 14, 2023

Reporting Summary

Nature Portfolio wishes to improve the reproducibility of the work that we publish. This form provides structure for consistency and transparency in reporting. For further information on Nature Portfolio policies, see our Editorial Policies and the Editorial Policy Checklist.

Statistics

For all statistical analyses, confirm that the following items are present in the figure legend, table legend, main text, or Methods section.

n/a	Confirmed	
☐	☒	The exact sample size (*n*) for each experimental group/condition, given as a discrete number and unit of measurement
☐	☒	A statement on whether measurements were taken from distinct samples or whether the same sample was measured repeatedly
☐	☒	The statistical test(s) used AND whether they are one- or two-sided<br>*Only common tests should be described solely by name; describe more complex techniques in the Methods section.*
☒	☐	A description of all covariates tested
☐	☒	A description of any assumptions or corrections, such as tests of normality and adjustment for multiple comparisons
☐	☒	A full description of the statistical parameters including central tendency (e.g. means) or other basic estimates (e.g. regression coefficient) AND variation (e.g. standard deviation) or associated estimates of uncertainty (e.g. confidence intervals)
☐	☒	For null hypothesis testing, the test statistic (e.g. *F*, *t*, *r*) with confidence intervals, effect sizes, degrees of freedom and *P* value noted<br>*Give P values as exact values whenever suitable.*
☒	☐	For Bayesian analysis, information on the choice of priors and Markov chain Monte Carlo settings
☒	☐	For hierarchical and complex designs, identification of the appropriate level for tests and full reporting of outcomes
☐	☒	Estimates of effect sizes (e.g. Cohen's *d*, Pearson's *r*), indicating how they were calculated

Our web collection on statistics for biologists contains articles on many of the points above.

Software and code

Policy information about availability of computer code

Data collection	Image Lab Software (Biorad v6.0.1), Omega V5.5 (BMG), BD FACStation 6.1 (BD Biosciences), StepOne v2.3 (Applied Biosystem)
Data analysis	Cyflogic (v1.2.1)<br>FlowJo (v10.8.1)<br>ggbeeswarm (v0.7.1 and above)<br>ggResidpanel (v0.3.0)<br>ggplot2 (v3.3.0 and above)<br>grafify (v2.0 and above)<br>GraphPad Prism (v8.0 and above)<br>Image Lab (v6.0.1)<br>lme4 (v1.1-25 and above)<br>lmerTest (v3.1-2 and above)<br>MARS (v4.01)<br>Microsoft Office 365 Enterprise<br>performance (v0.4.3 and above)<br>R (v4.0 and above)<br>RStudio Desktop (v1.3 and above)

For manuscripts utilizing custom algorithms or software that are central to the research but not yet described in published literature, software must be made available to editors and reviewers. We strongly encourage code deposition in a community repository (e.g. GitHub). See the Nature Portfolio guidelines for submitting code & software for further information.

Data

Policy information about availability of data

All manuscripts must include a data availability statement. This statement should provide the following information, where applicable:

- Accession codes, unique identifiers, or web links for publicly available datasets
- A description of any restrictions on data availability
- For clinical datasets or third party data, please ensure that the statement adheres to our policy

Source data are provided in the Source Data file. Further information and requests for resources and reagents should be directed to Avinash Shenoy (a.shenoy@imperial.ac.uk).

Human research participants

Policy information about studies involving human research participants and Sex and Gender in Research.

Reporting on sex and gender	N/A
Population characteristics	N/A
Recruitment	N/A
Ethics oversight	N/A

Note that full information on the approval of the study protocol must also be provided in the manuscript.

Field-specific reporting

Please select the one below that is the best fit for your research. If you are not sure, read the appropriate sections before making your selection.

☒ Life sciences ☐ Behavioural & social sciences ☐ Ecological, evolutionary & environmental sciences

For a reference copy of the document with all sections, see nature.com/documents/nr-reporting-summary-flat.pdf

Life sciences study design

All studies must disclose on these points even when the disclosure is negative.

Sample size	No statistical methods were used to pre-determine sample sizes. Group sizes were used based on previous experience and standards in the field.
Data exclusions	No data were excluded from the analyses.
Replication	All the experiments were reliably reproduced as validated by at least two independent experiments.<br>Several experiments were performed by two different investigators independently with similar results. All attempts of replication were successful.
Randomization	Experimental groups were randomized. Mice were grouped according to genotype and all experiments were performed with age- and sex-matched mice.
Blinding	Investigator was blinded during disease scoring of LPS treated mice (Fig 2H). For other experiments, investigators were not blinded because the investigator analysing data was also collecting it. Experimenters were blinded to siRNA in the RNAi screens until data analyses of three independent repeats.

Reporting for specific materials, systems and methods

We require information from authors about some types of materials, experimental systems and methods used in many studies. Here, indicate whether each material, system or method listed is relevant to your study. If you are not sure if a list item applies to your research, read the appropriate section before selecting a response.

Materials & experimental systems

n/a	Involved in the study	
☐	☒	Antibodies
☐	☒	Eukaryotic cell lines
☒	☐	Palaeontology and archaeology
☐	☒	Animals and other organisms
☒	☐	Clinical data
☒	☐	Dual use research of concern

Methods

n/a	Involved in the study	
☒	☐	ChIP-seq
☐	☒	Flow cytometry
☒	☐	MRI-based neuroimaging

Antibodies

Antibodies used

This list is in the following order:
Number
Antibody
Company
Catalogue number
Antibody dilution
1.
Anti-CD115-APC
Biolegend
Cat# 135509
1:100
2.
Anti- F4/80 Antigen APC
Tonbo Biosciences
Cat# 20-4801-U025
1:100
3.
Anti- GSDMD
Abcam
Cat# ab209845
1:1000
4.
Anti-ASC (AL177)
Adipogen
Cat# AG-25B-0006-C100
1:1000
5.
Anti-Caspase-1 (Casper1)
Adiopgen
Cat# AG-20B-0042-C100
1:1000
6.
Anti-Cd11b-FITC
Biolegend
Cat# 101206
1:100
7.
Anti-CD16/32
Biolegend
Cat# 101302
1:100
8.
Anti-c-Myc (9E10)
SCBT
Cat# sc-40
1:3000
9.
Anti-Flag M2 antibody
Sigma-Aldrich
Cat# F1804
1:2000
10.
Anti-FP (i.e., anti-GFP/mVenus/AcGFP) mouse monoclonal
Roche
Cat# 11814460001
1:2000
11.
Anti-GAPDH
Sant Cruz

Cat# sc-365062
1:2000
12.
Anti-IL-1β/IL-1F2 Goat polyclonal Antibody
R&D systems
Cat# AF401
1:2000
13.
Anti-Ly-6G-APC
Biolegend
Cat# 127614
1:100
14.
Anti-NLRP3 (Cryo2)
Adipogen
Cat# AG-20B-0014-C100
1:1000
15.
Anti-UBE2L3
GeneTex
Cat# GTX104717
1:3000
16.
Anti-UBE2L3 (B-11) FITC
Santa Cruz
Cat# sc-390032
1:100
17.
Anti-β-Actin–Peroxidase
Sigma-Aldrich
Cat# A3854
1:10000
18.
Donkey anti-Goat IgG(H+L) Secondary Antibody, HRP conjugate
Thermo Fisher Scientific
Cat# A16005
1:20000
19.
Donkey anti-Rabbit IgG (H+L) Secondary Antibody, HRP conjugate
Thermo Fisher Scientific
Cat# A16035
1:20000
20.
Goat anti-Mouse IgG (H+L) Highly Cross-Adsorbed Secondary Antibody HRP
Thermo Fisher Scientific
Cat# A16078
1:20000
21.
Normal mouse IgG2α FITC
Santa Cruz
Cat# sc-2856
1:100
22.
Rat IgG2α k -APC isotype
Biolegend
Cat# 400511
1:100

Validation

This list is in the following order:
Number
Antibody
Company
Antibody validation in the laboratory or by commercial vendor (text and links from websites).

1
Anti-CD115-APC
Biolegend
Each lot of this antibody is quality control tested by immunofluorescent staining with flow cytometric analysis. For flow cytometric staining, the suggested use of this reagent is ≤ 0.25 µg per 106 cells in 100 µl volume. It is recommended that the reagent be titrated for optimal performance for each application.
https://www.biolegend.com/en-us/products/apc-anti-mouse-cd115-csf-1r-antibody-6336?GroupID=BLG8949
2
Anti- F4/80 Antigen APC
Tonbo Biosciences
This antibody preparation has been quality-tested for flow cytometry using mouse spleen cells, or an appropriate cell type (where

indicated).
https://cytekbio.com/products/apc-anti-mouse-f4-80-antigen-bm8-1?variant=40581236424740
3
Anti- GSDMD
Abcam
Knockout validated
https://www.abcam.com/products/primary-antibodies/gsdmd-antibody-epr19828-ab209845.html
4
Anti-ASC (AL177)
Adipogen
We have validated this antibody against Asc-/- iBMDMs.
5
Anti-Caspase-1 (Casper1)
Adiopgen
We have validated against Casp1/11-/- iBMDMs.
Recognizes endogenous full-length and activated (p20 fragment) mouse caspase-1. Described to cross-react with full-length and activated (p20 fragment) of rat caspase-1 . https://adipogen.com/ag-20b-0042-anti-caspase-1-p20-mouse-mab-casper-1.html
6
Anti-Cd11b-FITC
Biolegend
Each lot of this antibody is quality control tested by immunofluorescent staining with flow cytometric analysis.
https://www.biolegend.com/en-us/products/fitc-anti-mouse-human-cd11b-antibody-347?GroupID=BLG10660
7
Anti-CD16/32
Biolegend
Each lot of this antibody is quality control tested by immunofluorescent staining with flow cytometric analysis.
https://www.biolegend.com/en-us/products/purified-anti-mouse-cd16-32-antibody-190
8
Anti-c-Myc (9E10)
SCBT
This Myc antibody is recommended for detection of c-Myc p67 and c-Myc tagged fusion proteins of mouse, rat, human, monkey, feline and canine origin by WB, IP, IF, IHC(P), FCM and ELISA; non cross-reactive with N-Myc or L-Myc proteins. Widely used in combination with eukaryotic expression vectors encoding proteins with c-Myc (amino acids 408-439) epitope tag.
https://www.scbt.com/p/c-myc-antibody-9e10
9
Anti-Flag M2 antibody
Sigma-Aldrich
Optimized for single banded detection of FLAG fusion proteins in mammalian, plant, and bacterial expression systems.
https://www.sigmaaldrich.com/GB/en/product/sigma/f1804
10
Anti-FP (i.e., anti-GFP/mVenus/AcGFP) mouse monoclonal
Roche
Anti-GFP is a mixture of two clones (7.1 and 13.1). Monoclonal antibody for detection of both wild-type and mutant forms of GFP or GFP fusions using:
Immunoprecipitation, western blots.
https://www.sigmaaldrich.com/GB/en/product/roche/11814460001
11
Anti-GAPDH
Sant Cruz
Molecular Weight of GAPDH: 37 kDa.
Positive Controls: Hep G2 cell lysate: sc-2227, HeLa whole cell lysate:
sc-2200 or A549 cell lysate: sc-2413.
https://www.scbt.com/p/gapdh-antibody-g-9?requestFrom=search
12
Anti-IL-1β/IL-1F2 Goat polyclonal Antibody
R&D systems
Detects mouse IL-1 beta /IL-1F2 in direct ELISAs and Western blots.
https://www.rndsystems.com/products/mouse-il-1beta-il-1f2-antibody_af-401-na
13
Anti-Ly-6G-APC
Biolegend
Each lot of this antibody is quality control tested by immunofluorescent staining with flow cytometric analysis.
https://www.biolegend.com/en-us/products/apc-anti-mouse-ly-6g-antibody-6115
14
Anti-NLRP3 (Cryo2)
Adipogen
We have validated this antibody against Nlrp3-/- cells.
15
Anti-UBE2L3
GeneTex
We have tested this antibody against knockout or siRNA transfected cells.
16
Anti-UBE2L3 (B-11) FITC
Santa Cruz
We have tested this antibody against knockout or siRNA transfected cells.
17

Anti-β-Actin–Peroxidase
Sigma-Aldrich
western blot: 1:25,000-1:50,000 using cell extracts of human foreskin fibroblasts or chicken fibroblasts.
https://www.sigmaaldrich.com/GB/en/product/sigma/a3854
18
Donkey anti-Goat IgG(H+L) Secondary Antibody, HRP conjugate
Thermo Fisher Scientific
Based on Immunoelectrophoresis, no reactivity is observed to: non-immunoglobulin goat serum immunoglobulins, IgG from human, mouse, rabbit or rat.
https://www.thermofisher.com/antibody/product/Donkey-anti-Goat-IgG-H-L-Cross-Adsorbed-Secondary-Antibody-Polyclonal/A16005
19
Donkey anti-Rabbit IgG (H+L) Secondary Antibody, HRP conjugate
Thermo Fisher Scientific
Based on Immunoelectrophoresis, no reactivity is observed to: non-immunoglobulin rabbit serum proteins, IgG from bovine, chicken, goat, guinea pig, hamster, horse, human, mouse, rat or sheep.
https://www.thermofisher.com/antibody/product/Donkey-anti-Rabbit-IgG-H-L-Highly-Cross-Adsorbed-Secondary-Antibody-Polyclonal/A16035
20
Goat anti-Mouse IgG (H+L) Highly Cross-Adsorbed Secondary Antibody HRP
Thermo Fisher Scientific
Based on Immunoelectrophoresis, no reactivity is observed to: non-immunoglobulin mouse serum proteins, bovine, goat, human, rabbit or rat IgG.
https://www.thermofisher.com/antibody/product/Goat-anti-Mouse-IgG-H-L-Highly-Cross-Adsorbed-Secondary-Antibody-Polyclonal/A16078
21
Normal mouse IgG2α FITC
Santa Cruz
recommended for use as a negative control immunoglobulin in place of a target specific primary antibody of the same isotype (mouse IgG2a) by IF, IHC(P) and FCM applications.
https://www.scbt.com/p/normal-mouse-igg2a-fitc?requestFrom=search
22
Rat IgG2α k -APC isotype
Biolegend
Each lot of this antibody is quality control tested by immunofluorescent staining with flow cytometric analysis as negative control.
https://www.biolegend.com/en-gb/products/apc-rat-igg2a-kappa-isotype-ctrl-1838

Eukaryotic cell lines

Policy information about cell lines and Sex and Gender in Research

Cell line source(s)	HEK293E (John MacMicking laboratory, Yale University) and immortalised murine bone marrow derived macrophages from previous work (Eldridge et al, 2017, Cell Reports 18, 1285–1297).
Authentication	STR typing with a commercial vendor.
Mycoplasma contamination	We routinely test cell lines for Mycoplasma contamination using PCR based kit, and found them negative.
Commonly misidentified lines (See ICLAC register)	No commonly misidentified cell lines were used.

Animals and other research organisms

Policy information about studies involving animals; ARRIVE guidelines recommended for reporting animal research, and Sex and Gender in Research

Laboratory animals	Mice were housed and bred in dedicated animal facilities of Imperial College London (12h light/dark cycle; 22+/-2°C; 30 to 40% humidity) . All mice were used on a C57BL/6J background. Eight to sixteen weeks-old male and female animals (C57BL/6, B6-Tg(Csf1r-cre/Esr1)1Jwp, and UBE2L3fx/fx) were used for all the experiments. Mice were housed and bred in dedicated animal facilities of Imperial College London (12h light/dark cycle; 22+/-2°C; 30 to 40% humidity). Mice were housed IVC cages with corn cob bedding and enrichments including nesting material, refuges and gnawing sticks. Mice were fed with RM1(E) rodent diet (SDS Diet, Dietex, UK) and water ad libitum.
Wild animals	No wild animals were used in this study.
Reporting on sex	The finding applies to both the sex, and eight to sixteen weeks-old male and female mice were used.
Field-collected samples	The study did not involve samples collected from the field.
Ethics oversight	All work with mice was performed in accordance with the Animals (Scientific Procedures) Act 1986 and was approved by the local ethics review committee at Imperial College London.

Note that full information on the approval of the study protocol must also be provided in the manuscript.

Flow Cytometry

Plots

Confirm that:

☒ The axis labels state the marker and fluorochrome used (e.g. CD4-FITC).

☒ The axis scales are clearly visible. Include numbers along axes only for bottom left plot of group (a 'group' is an analysis of identical markers).

☒ All plots are contour plots with outliers or pseudocolor plots.

☒ A numerical value for number of cells or percentage (with statistics) is provided.

Methodology

Sample preparation	Cells (1×10^6) collected from the peritoneal lavage of mice were washed in flow cytometry staining buffer and incubated with 1:100 dilution of anti-mouse CD16/32 (Fc block) antibody for 10 min at room temperature. Cell surface marker staining was performed using 1:100 dilution of respective antibodies (e.g., anti-mouse F4/80-APC, anti-mouse Ly6G-APC, or anti-mouse CD115 (Csf1r)-APC) in dark for 30 min at 4°C in staining buffer. Cells were washed thrice with staining buffer followed by fixation with IC fixation buffer for 15 min. For intracellular antigen staining, cells were washed thrice with permeabilization buffer and incubated with permeabilization buffer for 10 min at 4°C. UBE2L3 was stained using anti-mouse Ube2l3-FITC antibody at 1:100 dilution in dark for 30 min at 4°C in permeabilization buffer, followed by three washes in staining buffer. Total neutrophils were estimated by staining cells with anti-mouse Cd11b-FITC and anti-mouse Ly-6G-APC. Data was collected on a BD FACSCaliburTM and analysed with Cyflogic or FlowJo software.
Instrument	BD FACSCalibur
Software	BDFACStation 6.1 (BD Biosciences) for data collection; Cyflogic (CyFlo Ltd) and FlowJo 10.8 for analysis
Cell population abundance	This was a simple 2 color analysis and 100% of cells were included for analysis
Gating strategy	FSC-H vs. SSC-H density plot gating was performed to identify intact cells. Gate boundaries for APC tagged antibodies (CD115, Ly-6G, F4/80) and FITC tagged antibodies (UBE2L3, Cd11b) were defined based on cells stained with normal IgG2a isotype APC and normal IgG2a FITC respectively and then double positive cells were quantified.

☒ Tick this box to confirm that a figure exemplifying the gating strategy is provided in the Supplementary Information.
